# Epidemiological and Clinical Evidence on the Association Between Wildfire PM2.5 and Pulmonary Health Outcomes

**DOI:** 10.7759/cureus.91239

**Published:** 2025-08-29

**Authors:** Shaheen Rizly

**Affiliations:** 1 Internal Medicine, New York Presbyterian Brooklyn Methodist Hospital, New York City, USA

**Keywords:** asthma exacerbations, chronic lung disease, climate change, copd outcomes, ild progression, pm2.5 exposure, pulmonary health, smoke inhalation, wildfire air pollution, wildfire smoke

## Abstract

As climate change accelerates the frequency and severity of wildfires worldwide, the pulmonary consequences of wildfire smoke exposure have emerged as an important yet under-recognized public health concern. Wildfire-related fine particulate matter (PM2.5) possesses distinct toxicologic properties compared to urban PM2.5 and disproportionately affects vulnerable populations, including children, older adults, and individuals with chronic respiratory diseases. This narrative review synthesizes current epidemiological, mechanistic, and clinical evidence on the impact of wildfire PM2.5 on exacerbations and progression of asthma, chronic obstructive pulmonary disease (COPD), and interstitial lung disease (ILD), drawing from recent large-scale meta-analyses and international cohort studies. We reviewed data from over 30 peer-reviewed sources, including longitudinal cohorts, systematic reviews, environmental modeling studies, and public health guidelines. Special attention was given to pediatric populations, the elderly, and patients with pre-existing pulmonary conditions. Wildfire PM2.5 is consistently associated with significant increases in emergency department visits and hospitalizations for asthma and COPD, with relative risks often exceeding those of urban PM2.5. Pediatric asthma-related visits may increase by over 30% during wildfire events, while elderly COPD patients face heightened hospitalization and mortality risks. ILD patients, especially those with idiopathic pulmonary fibrosis (IPF), exhibit higher rates of acute exacerbations and accelerated disease progression with PM2.5 levels as low as 8-10 μg/m³. Mechanistically, wildfire PM2.5 promotes oxidative stress, epithelial injury, and immune dysregulation, contributing to disease exacerbation and chronic progression. Preventive interventions such as high-efficiency particulate air (HEPA) filtration, indoor sheltering, and respiratory protection significantly reduce PM2.5 exposure and related morbidity. Wildfire PM2.5 is a potent and growing contributor to pulmonary morbidity, with a higher toxicity profile than other ambient air pollutants. While acute impacts are well-documented, long-term consequences remain understudied. Urgent research is needed to define chronic health outcomes, develop interventions, and inform public health policy. As wildfires become more frequent and intense globally, clinicians must be prepared to recognize and manage the growing burden of wildfire-related pulmonary disease.

## Introduction and background

Short-term effects: asthma, COPD, and ILD exacerbations

Wildfire-related fine particulate matter (PM2.5) exposure is associated with acute exacerbations of chronic respiratory diseases, with the strongest evidence for asthma and chronic obstructive pulmonary disease (COPD), and emerging data for interstitial lung disease (ILD). Systematic reviews and meta-analyses consistently demonstrate that short-term increases in wildfire PM2.5 are linked to significant rises in emergency department (ED) visits and hospitalizations for asthma and COPD. For example, a meta-analysis found that each 10 μg/m³ increase in wildfire PM2.5 is associated with a 6% increase in asthma hospitalizations (relative risk {RR} 1.06, 95% CI: 1.02-1.09) and a 7% increase in asthma-related ED visits (RR 1.07, 95% CI: 1.04-1.09) [1,2]. These effects are more pronounced than those observed for urban PM2.5, likely due to the unique chemical composition and smaller particle size of wildfire smoke [2-4].

Children, especially those under five years of age, are particularly vulnerable. The American Academy of Pediatrics states that wildfire PM2.5 is up to 10 times more harmful to children’s respiratory health than PM2.5 from other sources, with the greatest impact in children younger than five years of age [5,6]. A large cohort study in California found that a 10-unit increase in wildfire PM2.5 was associated with a 30% increase in pediatric respiratory visits, compared to only a 3.7% increase for non-wildfire PM2.5 [6]. In Calgary, Canada, pediatric asthma exacerbations increased significantly during wildfire smoke events with an incidence rate ratio of 1.13 (95% CI: 1.02-1.24), an effect not seen with general air pollution [7]. The risk of respiratory-related healthcare visits is highest within the first three days of exposure, with the greatest risk on the day of exposure and persisting for several days [8,9].

Older adults with COPD are also at heightened risk. A large retrospective cohort study of over 10 million Medicare beneficiaries in the western United States found that increasing same-day and preceding week smoke PM2.5 from 0 to 40 μg/m³ was associated with an average increase of 2.40 respiratory hospitalizations per 100,000 individuals (95% CI: 0.17-4.63) [10]. Meta-analyses confirm that wildfire PM2.5 exposure is associated with increased risk of respiratory hospital admissions (RR: 1.04, 95% CI: 1.02-1.05 per 10 μg/m³ increase) and ED visits (RR: 1.04, 95% CI: 1.02-1.06), with the effect size notably higher for respiratory outcomes than for cardiovascular events [11]. Long-term exposure to wildfire PM2.5 is associated with a 9.2% increase in COPD mortality per 1 μg/m³ increment among elderly individuals (95% CI: 8.8-9.7%) [12].

ILD, particularly idiopathic pulmonary fibrosis (IPF) and hypersensitivity pneumonitis (HP), is less well studied but available evidence indicates that PM2.5 exposure is associated with increased risk of acute exacerbations and disease progression. A meta-analysis found a nearly twofold increased risk of acute exacerbations of IPF per 10 μg/m³ increment in PM2.5 (RR: 1.94, 95% CI: 1.30-2.90) [13]. A large multicenter cohort study demonstrated that each 1 μg/m³ increase in five-year time-varying PM2.5 exposure was associated with a hazard ratio of 1.09 (95% CI: 1.05-1.13) for mortality or transplant in fibrotic ILD [14]. The risk inflection point for adverse outcomes appears to be at PM2.5 concentrations above 8-10 μg/m³, emphasizing the importance of even slight increases in exposure for this population.

Comparative toxicity: wildfire PM2.5 versus urban PM2.5

Wildfire PM2.5 is consistently found to be more toxic per unit mass than urban PM2.5. This is attributed to its smaller particle size, higher oxidative and proinflammatory potential, and the presence of unique chemical constituents such as oxygenated polycyclic aromatic hydrocarbons, aldehydes, and nitrogen oxides [2-4]. Direct comparisons show that wildfire PM2.5 produces a disproportionately greater increase in asthma morbidity, particularly in children. For example, a study in Southern California found that a 10 μg/m³ increase in wildfire-specific PM2.5 was associated with a 1.3-10% increase in respiratory hospitalizations, compared to a 0.67-1.3% increase for non-wildfire PM2.5 [4]. The American Academy of Pediatrics highlights that PM2.5 from wildfire smoke is as much as 10 times more harmful to children’s respiratory health than PM2.5 from other sources [5,6]. Table 1 below provides a summary comparing the pulmonary health outcomes of wildfire-specific PM2.5 and urban PM2.5 across asthma, COPD, and ILD in both adult and pediatric populations [1-8].

**Table 1:** Highlights the greater toxicity and clinical impact of wildfire PM2.5, especially in vulnerable groups. COPD: chronic obstructive pulmonary disease; ILD: interstitial lung disease; PM: particulate matter

Disease/population	Wildfire PM2.5: pulmonary outcomes	Urban PM2.5: pulmonary outcomes	Relative toxicity/effect size	References
Asthma (children)	Marked increase in ED visits/hospitalizations; up to 10x more harmful; greatest impact in <5 years	Increased ED visits/hospitalizations, but lower effect size	Wildfire PM2.5 is much higher	[1,2,3,4,5]
Asthma (adults)	Increased exacerbations, ED visits, hospitalizations; higher risk per unit mass	Increased exacerbations, but lower risk per unit mass	Wildfire PM2.5 higher	[4,5,6,7]
COPD (older adults)	Increased exacerbations, hospitalizations, mortality; higher effect size	Increased exacerbations, but lower effect size	Wildfire PM2.5 higher	[4,5,7,8]
ILD (all ages)	Associated with exacerbation/progression; mechanistically more toxic, but direct comparative data are limited	Associated with exacerbation/progression	Wildfire PM2.5 is likely higher	[4,5,7]
General population	Stronger association with respiratory morbidity and mortality; higher risk in vulnerable groups	Increased risk, but lower effect size	Wildfire PM2.5 higher	[4,7,8]

Geographic and demographic patterns

The majority of high-quality epidemiological studies have been conducted in North America and Australia, where wildfire events are frequent and well-monitored [11,15-17]. In California, for example, the fraction of PM2.5-attributed health burden due to wildfire smoke has increased, offsetting public health gains from reductions in nonsmoke PM2.5 [17]. The highest relative risk and PM2.5-attributed burden for respiratory ED visits were observed in the oldest age groups, with notable disparities by race and geography. Rural, central, and northern California populations experienced the highest wildfire PM2.5-attributed burden rates, reflecting both increased exposure and underlying susceptibility [17].

Emerging data from South America and Southeast Asia confirm similar patterns. In Brazil, wildfire waves were associated with a 23% increase in respiratory hospital admissions nationwide, and a 38% increase in the Amazon region, with the greatest impact on children and older adults [18,19]. In southern Thailand, PM2.5 concentrations above 50 μg/m³ were associated with odds ratios for asthma exacerbations ranging from 1.41 to 1.64, with children aged 6-17 years being particularly susceptible even at moderate PM2.5 concentrations [20]. These findings underscore the global relevance of wildfire PM2.5 as a driver of respiratory morbidity.

## Review

Pathophysiologic mechanisms: oxidative stress, inflammation, and immune dysregulation

Wildfire PM2.5 is generated by the combustion of biomass and, in the wildland-urban interface, anthropogenic materials. Compared to urban PM2.5, wildfire PM2.5 contains a higher proportion of ultrafine particles, increased oxidative and proinflammatory chemical constituents, and a greater oxidative potential [16,21]. The small size and chemical composition of wildfire PM2.5 facilitate deep penetration into the distal airways and alveoli, where particles generate reactive oxygen species (ROS) and disrupt the airway epithelial barrier [16,21,22]. This leads to increased permeability, impaired mucociliary clearance, and direct cytotoxicity, initiating downstream inflammatory cascades.

Inhaled wildfire PM2.5 activates airway macrophages, dendritic cells, and epithelial cells, resulting in the release of proinflammatory cytokines (e.g., IL-1β, IL-6, TNF-α) and chemokines that recruit neutrophils and other immune cells [16,21,22]. Neutrophilic airway inflammation is a consistent finding in both human and animal studies of wildfire smoke exposure, and is associated with worsened lung function and increased airway hyperresponsiveness [23]. Wildfire PM2.5 can also modulate both innate and adaptive immune responses, enhancing Th2 and Th17 polarization, promoting allergic sensitization, and driving steroid-resistant airway inflammation [21,22].

Disease-specific mechanisms and age-related susceptibility

In asthma, wildfire PM2.5 exposure leads to increased airway hyperreactivity, mucus production, and bronchoconstriction, resulting in acute exacerbations [2,5,6,15,22]. Children are particularly vulnerable due to higher minute ventilation relative to body size, ongoing lung development, and immature antioxidant and immune defenses [5,6,8,24]. In COPD, wildfire PM2.5 exacerbates chronic airway inflammation, increases mucus production, and impairs host defense, leading to increased risk of acute exacerbations and respiratory failure [15,25]. Older adults with COPD are especially susceptible due to age-related declines in mucociliary clearance, antioxidant capacity, and immune regulation [15,25].

In ILD, particularly IPF and HP, repeated or high-intensity wildfire PM2.5 exposure may contribute to persistent epithelial injury, dysregulated repair, and promotion of fibrogenic pathways, thereby accelerating disease progression [13,26,27]. The pathophysiologic mechanisms are broadly similar across age groups and disease types but are modulated by developmental, immunologic, and disease-specific factors that influence susceptibility and clinical outcomes [16,21,22].

Asthma control and progression

Repeated exposure to wildfire PM2.5 is associated with persistent and potentially cumulative adverse effects on asthma control in both children and adults. While acute exposure is linked to increased rates of asthma exacerbations, there is growing concern that chronic or repeated exposure may contribute to airway remodeling, increased bronchial hyperresponsiveness, and a higher baseline level of airway inflammation, potentially leading to more severe or less controllable asthma over time [5,6,15,28]. The American Thoracic Society notes that few studies have evaluated the chronic health effects of repeated smoke exposure across wildfire seasons, but expert consensus supports the plausibility of persistent impairment in lung capacity and increased asthma severity with ongoing exposure [15,28].

COPD progression and mortality

For individuals with COPD, repeated wildfire PM2.5 exposure contributes significantly to disease progression and poorer health outcomes. Acute exposure is consistently associated with increased rates of COPD exacerbations, hospitalizations, and all-cause mortality, with the effect size often exceeding that of urban PM2.5 [15,25]. Long-term exposure to wildfire PM2.5 is associated with a 9.2% increase in COPD mortality per 1 μg/m³ increment among elderly individuals [12]. The pathophysiologic mechanisms underlying these effects include persistent oxidative stress, chronic airway and systemic inflammation, and impaired mucociliary clearance, all of which are potentiated by the chemical composition of wildfire PM2.5 [16,25].

ILD development and exacerbation

The relationship between wildfire PM2.5 exposure and ILD, including IPF and HP, is less well characterized but is an area of active investigation. Available evidence indicates that ambient PM2.5 exposure, regardless of source, is associated with increased risk of ILD exacerbation, progression, and mortality [13,26,27]. Wildfire PM2.5, with its higher oxidative and proinflammatory potential, is hypothesized to be particularly injurious; however, direct studies on ILD incidence and exacerbation are limited [13,26,27]. The risk inflection point for poor outcomes in fibrotic ILD appears to be at PM2.5 concentrations above 8-10 μg/m³ [14].

Research gaps in long-term outcomes

Despite the strong mechanistic and acute epidemiological evidence, there is a lack of large, well-characterized longitudinal studies or registries that specifically track long-term pulmonary outcomes such as new-onset asthma, COPD progression, or ILD incidence in populations repeatedly exposed to wildfire PM2.5 [15,28]. Most available studies are cross-sectional or focus on short-term outcomes, leaving the long-term trajectory of respiratory health in the context of recurrent wildfire exposure largely uncharacterized [28].

Diagnostic approaches

Diagnosis of wildfire smoke-related morbidity relies on a high index of suspicion during wildfire events, particularly in patients with pre-existing respiratory disease who present with acute symptom exacerbation [5,15]. For symptomatic individuals, clinicians should assess for acute exacerbations using standard clinical criteria, including history, physical examination, and, when indicated, spirometry or peak flow measurements [5,15]. In children, vigilance is required as wildfire PM2.5 is associated with a higher risk of asthma exacerbations and ED visits, particularly in those under five years of age [5,6,8].

Preventive interventions: exposure reduction

The consensus across guidelines from the American Thoracic Society, the American Academy of Pediatrics, and the American Heart Association is that exposure reduction is the cornerstone of prevention for all vulnerable groups [5,15,29,30]. The most effective intervention for reducing indoor PM2.5 is the use of high-efficiency particulate air (HEPA) filters or portable air cleaners (PACs). Systematic reviews report that HEPA filters can reduce indoor PM2.5 concentrations by 54-92%, with the American Thoracic Society specifically recommending their use in homes, schools, and offices [15,30]. Upgrading Heating, Ventilation, and Air Conditioning (HVAC) systems to use filters with a minimum efficiency reporting value (MERV) of 12 or higher can achieve reductions in PM2.5 of up to 87% [30].

Staying indoors with windows and doors closed during wildfire smoke events is universally recommended, especially for children and older adults [5,15,30]. Clean air shelters (public buildings equipped with advanced filtration) are a reliable community-level intervention, though their effectiveness during wildfire events has not been sufficiently tested in large-scale studies [30]. Relocation to areas with better air quality may be necessary during severe or prolonged smoke events, particularly for those with severe asthma, COPD, or ILD [5].

The use of N95 or P100 respirators is recommended for adults who must be outdoors during smoke events, as these masks can filter up to 94% of PM2.5 in single-pass testing [30]. However, N95 respirators do not fit young children, and medical and cloth masks offer limited or no protection against fine particulates [5,31]. Prolonged use of N95 masks may be associated with increased work of breathing and discomfort, particularly in hot weather, and they should be used with caution in individuals with underlying respiratory or cardiac disease [15].

Therapeutic strategies

For patients with pre-existing respiratory diseases, the primary therapeutic approach during wildfire smoke exposure is the optimization of baseline disease management. For asthma and COPD, this includes ensuring that patients have an updated action plan, access to rescue and controller medications, and clear instructions for medication adjustment during exacerbations [5,15]. Inhaled corticosteroids and other controller medications should be continued as prescribed, and short-acting bronchodilators should be readily available for acute symptoms [5,15]. For patients with ILD, similar principles apply, with an emphasis on early recognition of exacerbations and initiation of rescue therapy or escalation of care as needed [15,27].

A cost-effectiveness analysis in British Columbia projected that HEPA filter use could prevent thousands of asthma exacerbations, hundreds of emergency visits, and hospitalizations over a five-year period, with the intervention being cost-effective in most regions when government rebates were provided [32]. The American Academy of Pediatrics highlights that these interventions are particularly important for children under five, who are at greatest risk for severe outcomes [5,32].

Communication and health equity

Effective communication is important for ensuring uptake of preventive measures. The American Thoracic Society and systematic reviews highlight that messages delivered by trusted clinicians are more likely to result in behavioral change than passive public health messaging [15,33,34]. Communication strategies should be specified to the needs of culturally and linguistically diverse populations, as well as those with disabilities, to address disparities in access to information and resources [5,33,34]. Socioeconomic factors influence the ability to implement recommended interventions, and public health planning should prioritize support for disadvantaged communities [5,15,17,34].

Source apportionment and personal exposure assessment

Recent advances in source apportionment and personal exposure assessment for wildfire PM2.5 have significantly improved the precision and clinical relevance of risk estimation for chronic respiratory diseases. Ensemble-based statistical and machine learning models now combine satellite-derived aerosol optical depth, fire emissions inventories, atmospheric dispersion models, and meteorological data to generate high-resolution, source-specific PM2.5 estimates [35,36]. Personal monitoring devices provide high time-resolution data and can differentiate between biomass burning and fossil fuel combustion sources, offering insights into exposure profiles for specific tasks and populations [37]. The adoption of human mobility data into exposure assessment models further improves the accuracy of individual exposure estimates and facilitates the identification of environmental justice concerns [38].

Research gaps and future directions

Despite these advances, significant research gaps remain. There is a lack of large, prospective, population-based cohort studies with detailed exposure assessment and long-term follow-up to assess the development of new-onset asthma, COPD progression, or ILD incidence in populations repeatedly exposed to wildfire PM2.5 [15,28]. Most available studies are cross-sectional or focus on short-term outcomes, leaving the long-term trajectory of respiratory health in the context of recurrent wildfire exposure largely uncharacterized [28]. There is also a need for improved source apportionment techniques to distinguish wildfire PM2.5 from other sources, enabling more precise attribution of health effects and better targeting of interventions [36-38].

Clinical and public health recommendations

Based on the current evidence, the following recommendations are immediately actionable for clinicians and public health practitioners: for children with asthma, especially those under five years of age, and for older adults with COPD, exposure to wildfire PM2.5 should be minimized through the use of HEPA filtration (or MERV12+ HVAC filters), staying indoors with windows and doors closed, and, for adults, the use of N95 respirators when outdoor exposure is unavoidable [5,15,30,32]. These interventions are associated with significant reductions in asthma and COPD exacerbations, emergency visits, and hospitalizations, with the greatest benefit observed in children under five years and older adults with chronic respiratory disease [5,15,32]. For patients with ILD, clinicians should maintain a high index of suspicion for environmental contributions during wildfire events and prioritize exposure reduction [15,26,27].

Clinicians should ensure that patients with chronic respiratory diseases have updated action plans, access to rescue and controller medications, and clear instructions for medication adjustment during exacerbations [5,15]. Inhaled corticosteroids and other controller medications should be continued as prescribed, and short-acting bronchodilators should be readily available for acute symptoms [5,15]. For patients with ILD, early recognition of exacerbations and initiation of rescue therapy or escalation of care are essential [15,27].

Public health communication should be tailored, trusted, and accessible, with special attention to the needs of culturally and linguistically diverse and socioeconomically disadvantaged populations [5,15,33,34]. Public health planning should prioritize support for disadvantaged communities, including the provision of free or subsidized air filtration devices, targeted communication, and the establishment of accessible clean air shelters [5,15,17,34].

## Conclusions

Wildfire-related PM2.5 exposure is a potent and increasingly prevalent driver of acute and chronic respiratory morbidity, with effects that are more severe than those of urban PM2.5. The acute risk is highest within days of exposure, with children under five years and older adults with COPD being particularly vulnerable. While the long-term effects are less well characterized, there is growing concern about the potential for persistent respiratory morbidity with repeated exposure, especially for asthma control, COPD progression, and ILD development or exacerbation. The evidence base is strongest for North America and Australia, with emerging data from Southern Europe, South America, and Southeast Asia supporting similar conclusions. The American Thoracic Society, the American Academy of Pediatrics, and the American Heart Association all underscore the importance of exposure mitigation and targeted management for at-risk populations, while highlighting the need for further research into the long-term health effects of repeated wildfire smoke exposure and the effectiveness of community-level interventions. As wildfire activity continues to increase globally, ongoing refinement and standardization of exposure assessment, as well as the development of large, prospective cohort studies, will be essential for advancing both clinical care and epidemiological research in respiratory health.
